# Supplementary material for: Modulation of the Endomembrane System by the Anticancer Natural Product Superstolide/ZJ-101
Source: Int J Mol Sci. 2023 May 31;24(11):9575. doi: 10.3390/ijms24119575 (PMC10253484; doi:10.3390/ijms24119575)
Supplement: Supplementary file 1 [file ijms-24-09575-s001.zip › ijms-2414433-supplementary.pdf]

Modulation of the Endomembrane System by the Anticancer Natural Product Superstolide/ZJ-101

Phillip R. Sanchez[1,2], Sarah A. Head[2], Shan Qian[3], Haibo Qiu[3], Avishek Roy[3], Zhendong Jin[3], Wei Zheng[1*], Jun O. Liu[2]*

1 National Center for Advancing Translational Sciences (NCATS), NIH, Rockville, Maryland, USA

2 Department of Pharmacology & Molecular Sciences, Johns Hopkins School of Medicine, Baltimore, Maryland, USA

3 Department of Pharmaceutical Sciences and Experimental Therapeutics, College of Pharmacy, The University of Iowa, Iowa City, USA

* Correspondence: JOL, joliu@jhu.edu; WZ, wzheng@mail.nih.gov

Table of Contents

Supplementary Figure S1. Proliferation assays 2

Supplementary Figure S2. Golgi and lectin assays 3

Supplementary Figure S3. Additional transcriptome data 4

Supplementary Figure S4. Cellular Context 5

Supplementary Figure S5. Glycomics MS trace data 6

A

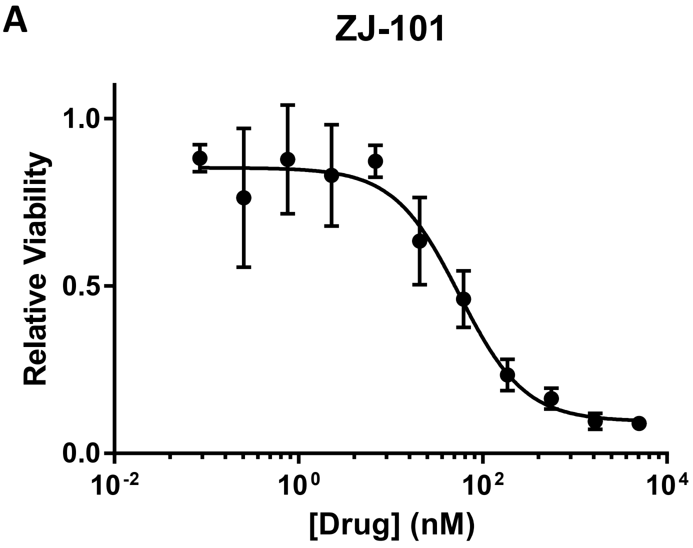

EC50	34.06 to 90.93

B

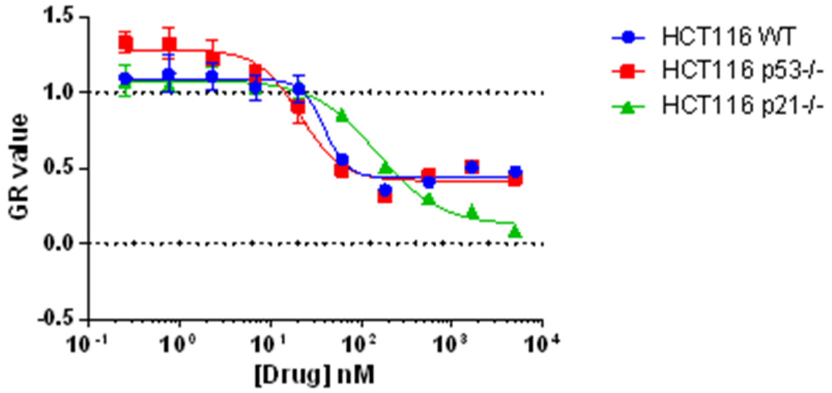

	HCT116 WT	HCT116 p53-/-	HCT116 p21-/-
EC50	28.48 to 55.10	15.17 to 29.84	115.4 to 197.4

Figure S1. Proliferation Assays (**A**) Resazurin dye viability assay of 72 h ZJ-101 treatment in MDA-MB-231 cells. (**B**) GR-assay of isogenic HCT116 cell lines with ZJ-101 treatment over 72 h.

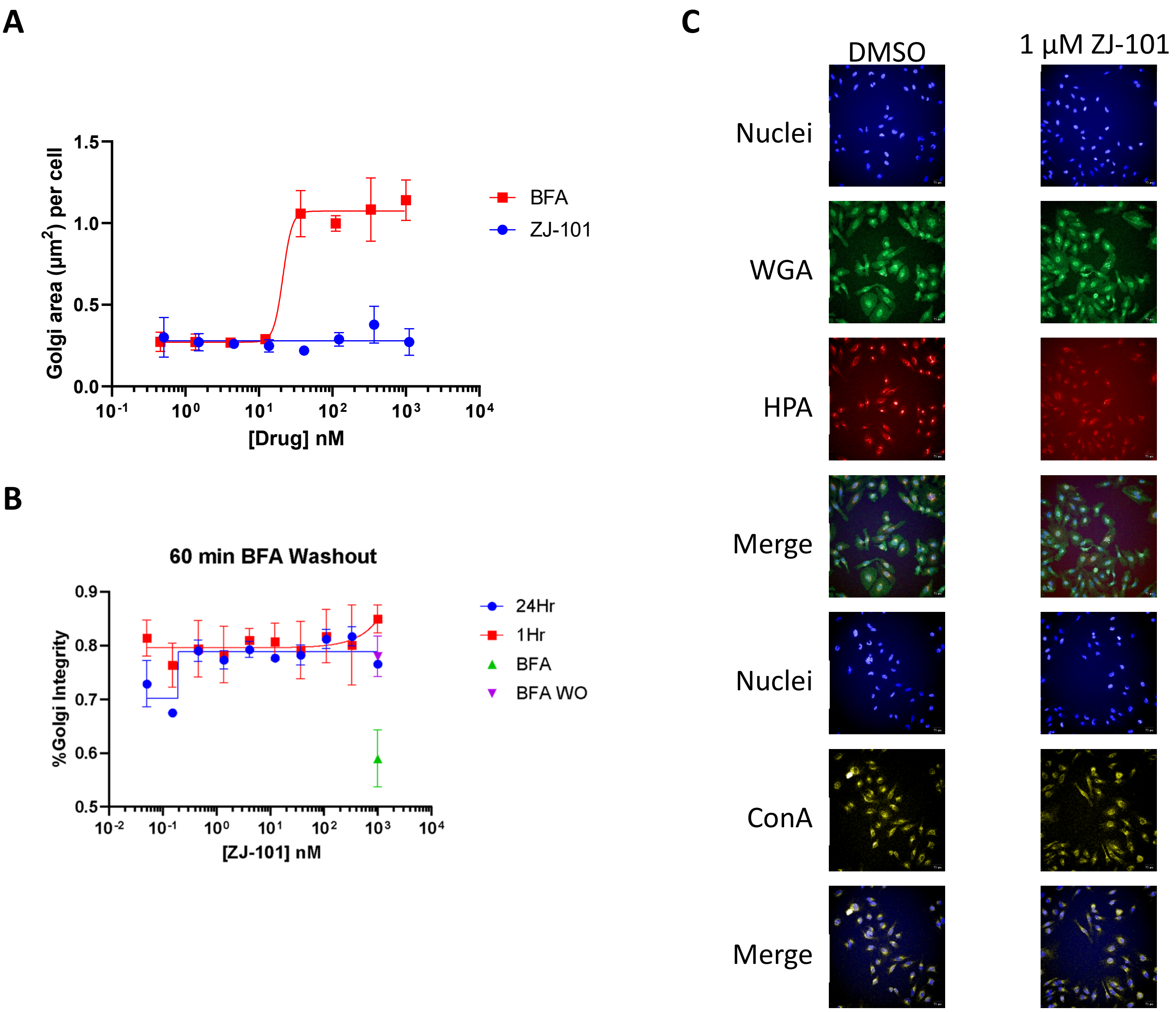

Figure S2. Golgi and Lectin Assays (**A**) Quantitation of dose-response effect of Brefeldin A (BFA) and ZJ-101 on GM130 stained golgi structure area. ZJ-101 does not lead to a redistribution of GM130 to the endoplasmic reticulum. (**B**) BFA washout experiment with indicated doses of ZJ-101 either at 1 hour or 24 hours of treatment prior to 60 min BFA pulse. (**C**) Representative images of ZJ-101 dose response fluorescent conjugate Lectin staining assay with WGA-488, HPA-647 and ConA-488 (pseudocolored yellow) with 1 uM ZJ-101 shown.

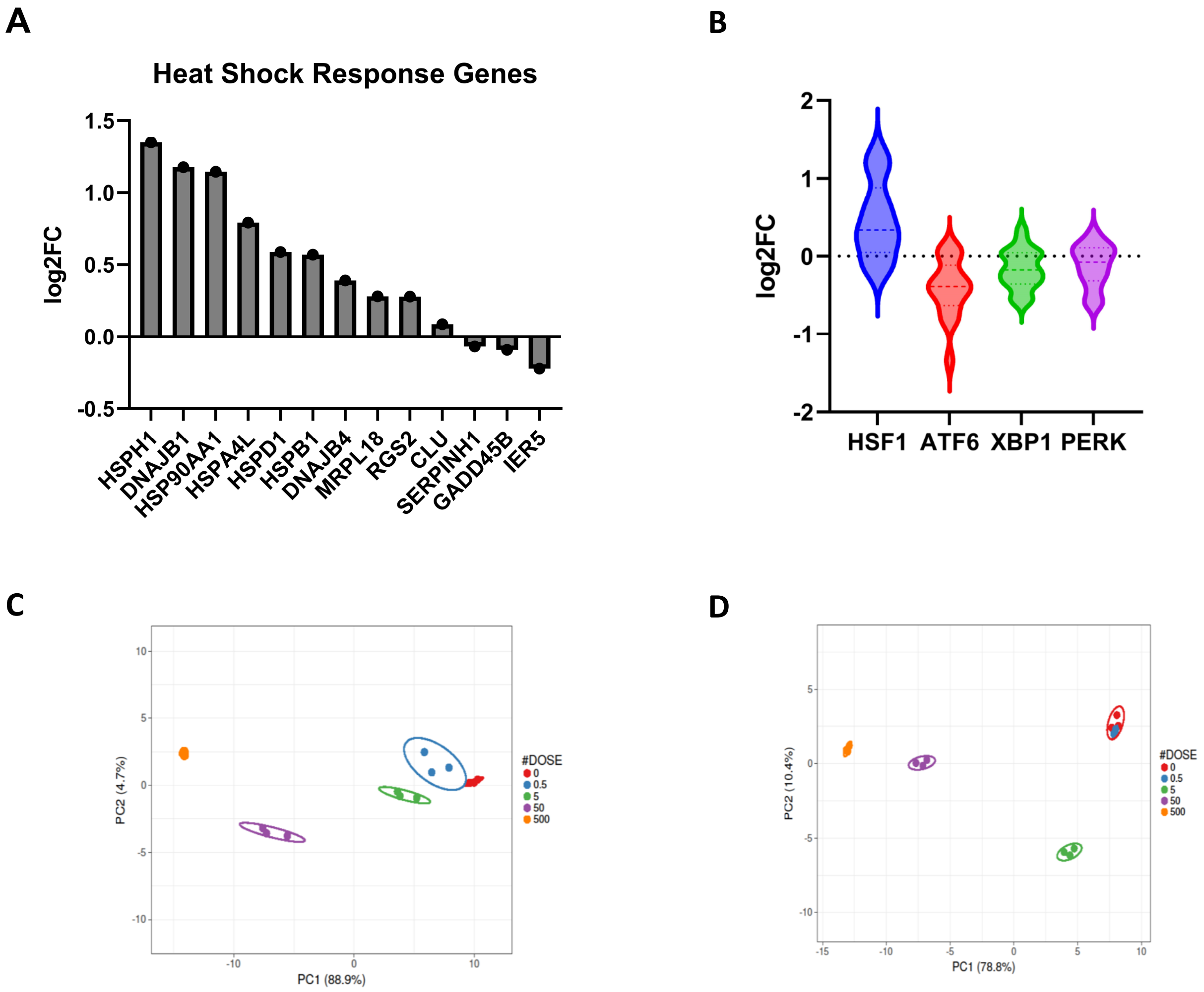

Figure S3. Additional transcriptome data (**A**) Curated heat shock genes from the 6 Hr 500 nM treatment of ZJ-101 in 2D format shows significant increases in heat shock genes such as HSPH1, DNAJB1 and HSP90AA1 (**B**) Geneset analysis of target genes from each of the arms of the Unfolded Protein Response (UPR) indicate little to no upregulation of XBP1 or PERK target genes with moderate decreases in ATF6 target genes. HSF1 responsive genes were increased relative to controls. (**C**) PCA plot for 6Hr ZJ-101 dose response in 2D cultured MDA-MB-231 cells. Transcript counts tables of the top 100 genes ordered by significance were processed and uploaded to ClustVis for principal component analysis. (**D**) PCA plot for 24 Hr ZJ-101 dose response in 3D cultured MDA-MB-231 cells. Transcript counts tables of the top 100 genes ordered by significance were processed and uploaded to ClustVis for principal component analysis.

Overnight treatment (i.e > 12 h) with ZJ-101 results in dislodged cells in 2D culture format as seen in Figure 1. Cell rounding can be observed at shorter time-points at high doses (> 1uM).

24 h treatment of pre-formed spheroids causes moderate cell adhesion loss as seen in Figure 2C. Spheroids can be fully dissolved at high doses (> 1uM) at this time-point.

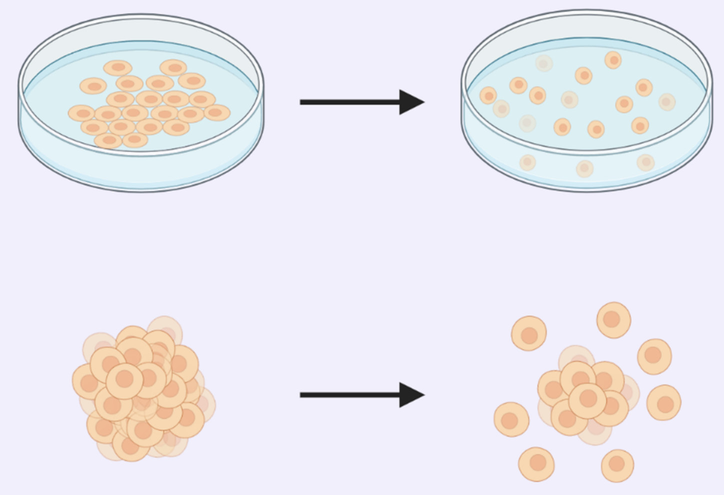

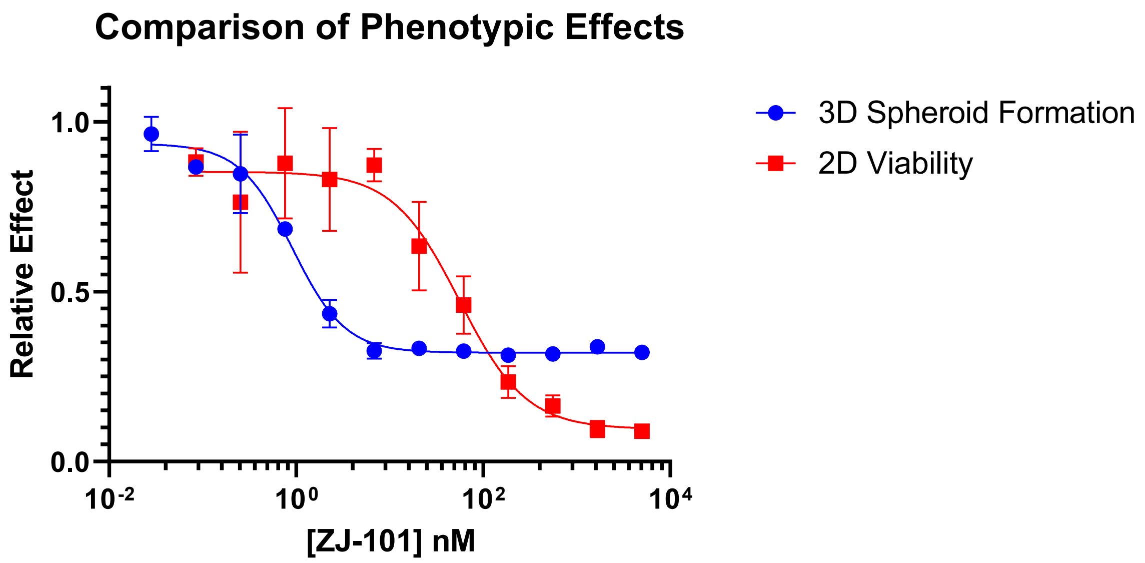

	3D Spheroid Formation	2D Viability
IC50	0.6926 to 1.162	33.98 to 96.62

Figure S4. Cellular Context. It is important to note that the difference in EC50's between the anti-proliferative and anti-adhesive effects is slightly larger than one order of magnitude. Therefore, transcriptomics experiments were performed using four logs of dose from 0.5 nM (below the 3D anti-adhesive EC50) to 500 nM (above the 2D anti-proliferative EC50) with careful attention paid to the above time-points when these doses resulted in morphological changes.

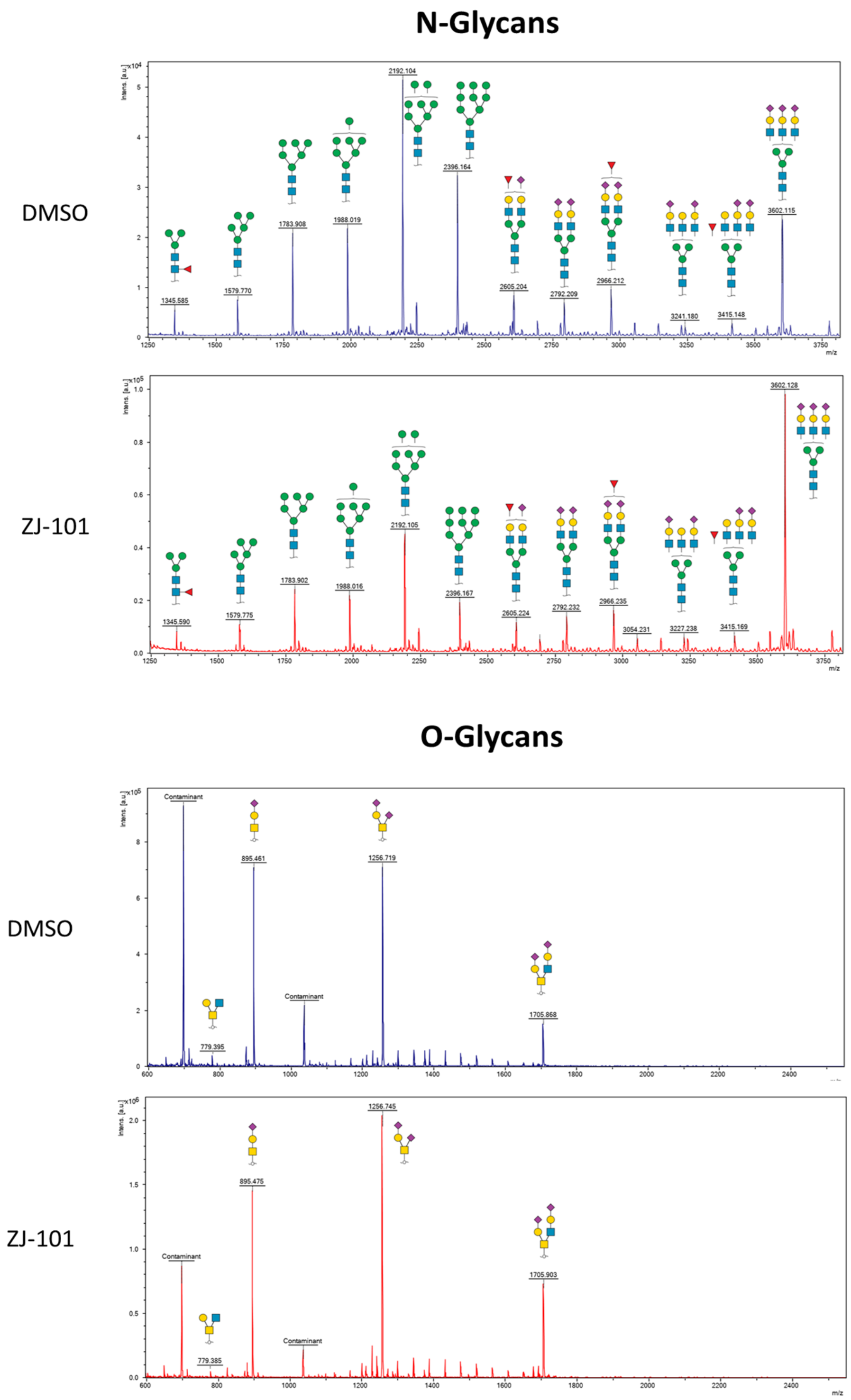

Figure S5. Glycomics MS trace data.
